# Bio-mechanical effects of femoral neck system versus cannulated screws on treating young patients with Pauwels type III femoral neck fractures: a finite element analysis

**DOI:** 10.1186/s12891-023-07110-5

**Published:** 2024-01-20

**Authors:** Xiao Fan, Yimin Zhou, Shiyou Dai, Kecheng Lao, Qiliang Zhang, Tengbo Yu

**Affiliations:** 1 Qingdao Municipal Hospital, Qingdao, Shandong 266011 China; 2 Dongzhimen Hospital of Beijing University of Traditional Chinese Medicine, Beijing, 100007 China

**Keywords:** Femoral neck fractures, Femoral neck system, Cannulated screws, Finite element analysis, Biomechanics

## Abstract

**Introduction:**

As a novel internal fixation for femoral neck fractures, the femoral neck system has some advantages for young Pauwels type III femoral neck fractures without clear biomechanical effects and mechanisms. Thus, the objection of the study is to realize the biomechanical effects and mechanism of FNS cannulated screws on treating young patients with Pauwels type III femoral neck fractures compared to cannulated screws which are commonly used for femoral neck fractures by finite element analysis.

**Methods:**

Firstly, the model of young Pauwels type III femoral neck fractures, femoral neck system (FNS), and three cannulated screws (CS) arranged in an inverted triangle were established, and the internal fixations were set up to fix young Pauwels type III femoral neck fractures. Under 2100 N load, the finite element was performed, and the deformation, peak von Mises stress (VMS), and contact at fracture segments were recorded to analyze the biomechanical effects and mechanism of FNS and three-CS fixing young Pauwels type III femoral neck fractures.

**Results:**

Compared to three-CS, the deformation of the whole model, internal fixation, and fracture segments after FNS fixation were lower, and the peak VMS of the whole model and the internal fixation after FNS were higher with lower peak VMS of the distal femur and the fracture segments. With a sticking contact status, the contact pressure at fracture segments after FNS fixation was lower than that of three-CS.

**Conclusions:**

FNS can provide better mechanical effects for young patients with Pauwels type III femoral neck fractures, which may be the mechanical mechanism of the clinical effects of FNS on femoral neck fracture. Although there is high stress on FNS, it is still an effective and safe internal fixation for young patients with Pauwels type III femoral neck fractures.

## Introduction

As the most common hip fractures in clinics, femoral neck fractures account for about 50% of hip fractures [1]. According to statistics [2], about 160,000 people occurred to femoral neck fractures every year, and most of them are elderly women with osteoporosis. With high morbidity and mortality, femoral neck fractures take a heavy economic burden on patients and society. So, how to treat femoral neck fractures effectively is a medical challenge. For elderly patients, femoral neck fractures are mostly caused by low-energy violence, and hip replacement surgery is thought to be the optimum treatment [3, 4]. While for young patients, femoral neck fractures are mostly caused by high-energy trauma, such as traffic accidents and high fall injuries, and internal fixation is the first choice [5]. Nevertheless, according to the Pauwels classification, most femoral neck fractures happened to young patients caused by high-energy trauma are Pauwels type III fractures which are extremely unstable with high shear force and shear stress leading to many complications such as femoral head necrosis, bone nonunion, internal fixation failure, and displacement. According to the previous studies [6–8], the incidence of bone nonunion and femoral head necrosis in young patients with femoral neck fractures was about 16–59% and 11–86% separately. Thus, it is challenging to treat young patients with Pauwels type III femoral neck fractures effectively.

Currently, three cannulated screws (3-CSs) arranged in an inverted triangle are commonly used to treat young patients with Pauwels type III femoral neck fractures. Although 3-CSs have the ability of compression and rotation resistance, many studies [9, 10] have reported that 3-CSs may cause a high risk of femoral neck shortening, coxa varus, femoral head necrosis, and inter fixation failure for Pauwels type III femoral neck fractures.

**Fig. 1:**
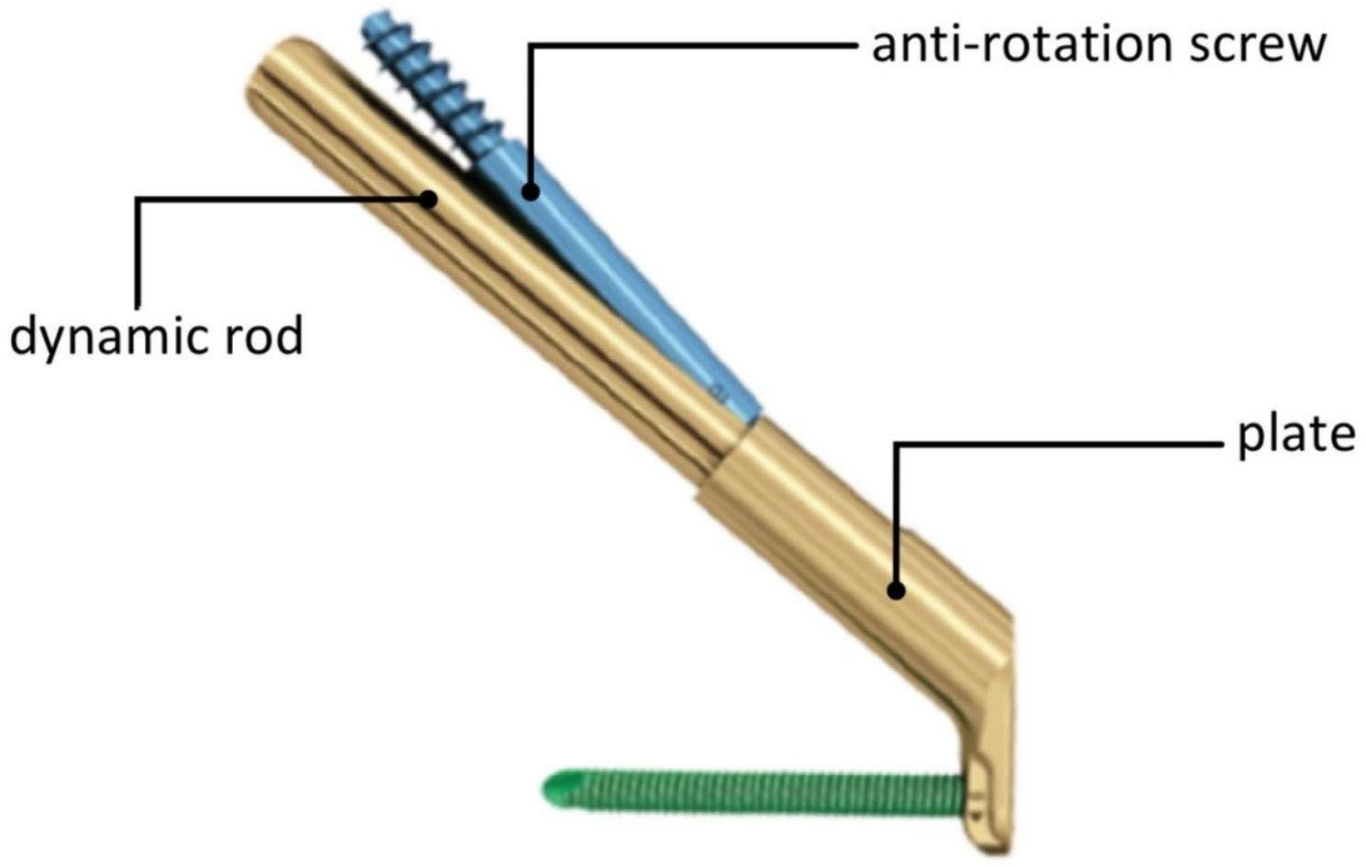
Physical picture of FNS

**Fig. 2:**
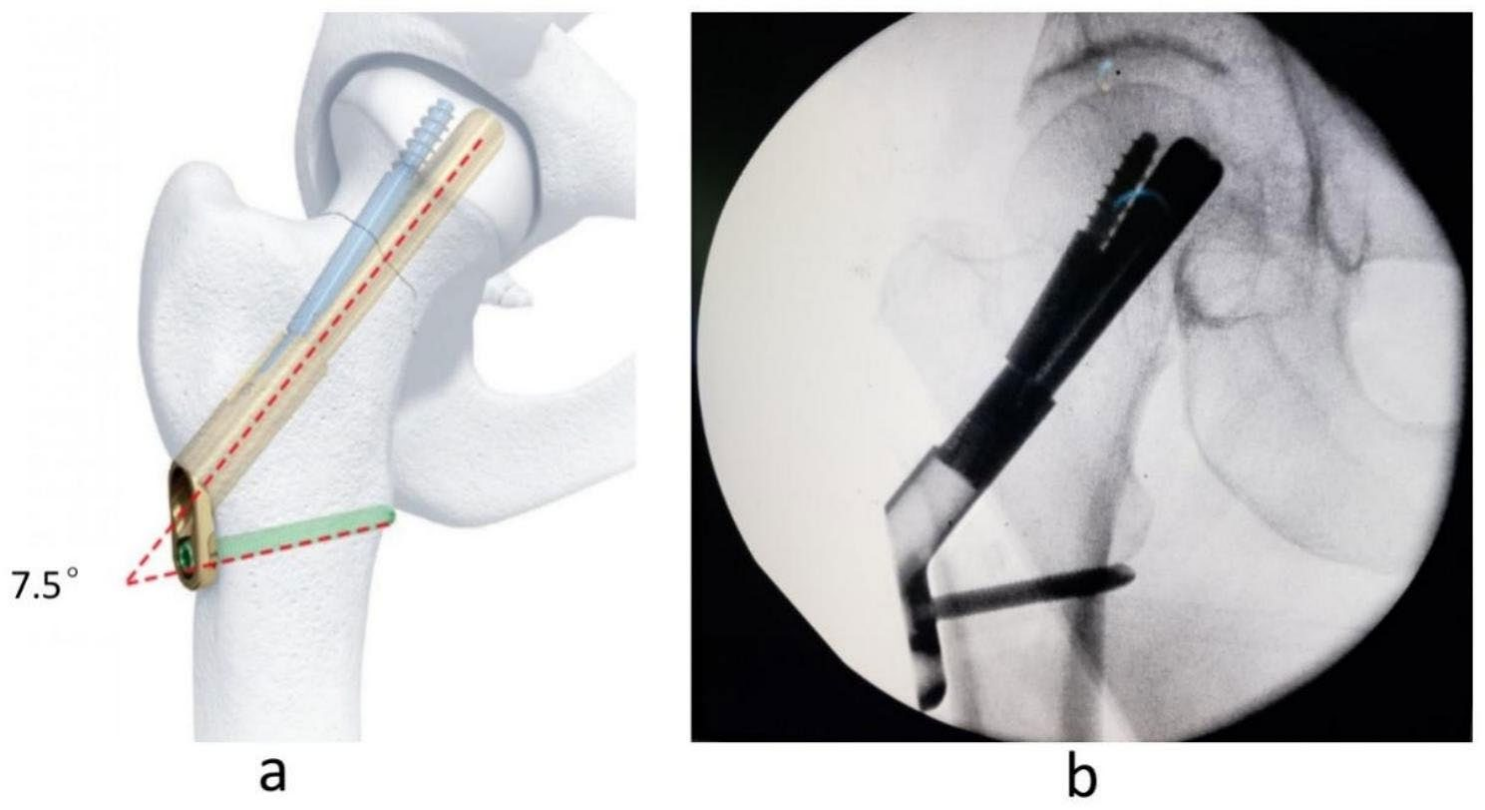
FNS fixing femoral neck fracture. (**a**) FNS fixing femoral neck fracture with 7.5 degrees included angle between plate and locking screw providing angle stability. (**b**) X-ray of FNS fixing femoral neck fracture intro-operation

Femoral neck system (FNS), a newly developed internal fixation device, consists of a dynamic rod (sliding screw), anti-rotation screw, and plate (Fig. 1) and was designed by DePuySynthes to treat femoral neck fractures (Fig. 2) in clinics in recent years with some mechanical advantages to resist shear force and shear stress [11]. So, it is thought to be an appropriate internal fixation device for young patients with Pauwels type III femoral neck fractures. However, there also are some failure cases of FNS for femoral neck fractures in clinics. So, there is still a debate about the optimal internal fixation device for young patients with Pauwels type III femoral neck fractures. Some clinical studies [12, 13] have reported that the effects of FNS treating femoral neck fractures are better than 3-CSs with a low risk of bone nonunion, femoral head necrosis, femoral neck shortening, and internal fixation failure. However, there is less systematical biomechanical analysis about FNS versus cannulated screws fixing Pauwels type III femoral neck fractures in young patients. Therefore, we performed the finite element analysis to compare the bio-mechanical effects of FNS and 3-CSs in treating young patients with Pauwels type III femoral neck fractures.

## Materials and methods

### The construction of Pauwels type III femoral neck fractures model

This research has been approved by the IRB of the authors’ affiliated institutions (The Qingdao Municipal Hospital, Approval Case No. 2015-22). The Femoral computed tomography (CT) data through a 64-slice CT scanner (SIEMENS, Germany) were obtained from a 25-year-old male volunteer who had signed the informed consent, and the files of Digital Imaging and Communications in Medicine (DICOM) were imported into Mimics 21.0 (Materialise, Belgium) to construct the 3D model of the femur that was exported in stereolithography (STL) format. Then, these files were imported into Geomagic Wrap 2017 software (Geomagic, USA) to perform smoothing, meshing, noise reduction, and surface fitting. Subsequently, the files were loaded into SolidWorks 2017 software (Dassault, France), and the 3D model of cortical bone and cancellous bone of the femur were constructed by Boolean operations. Finally, the 3D model of the femur of a young male was built successfully.

**Fig. 3:**
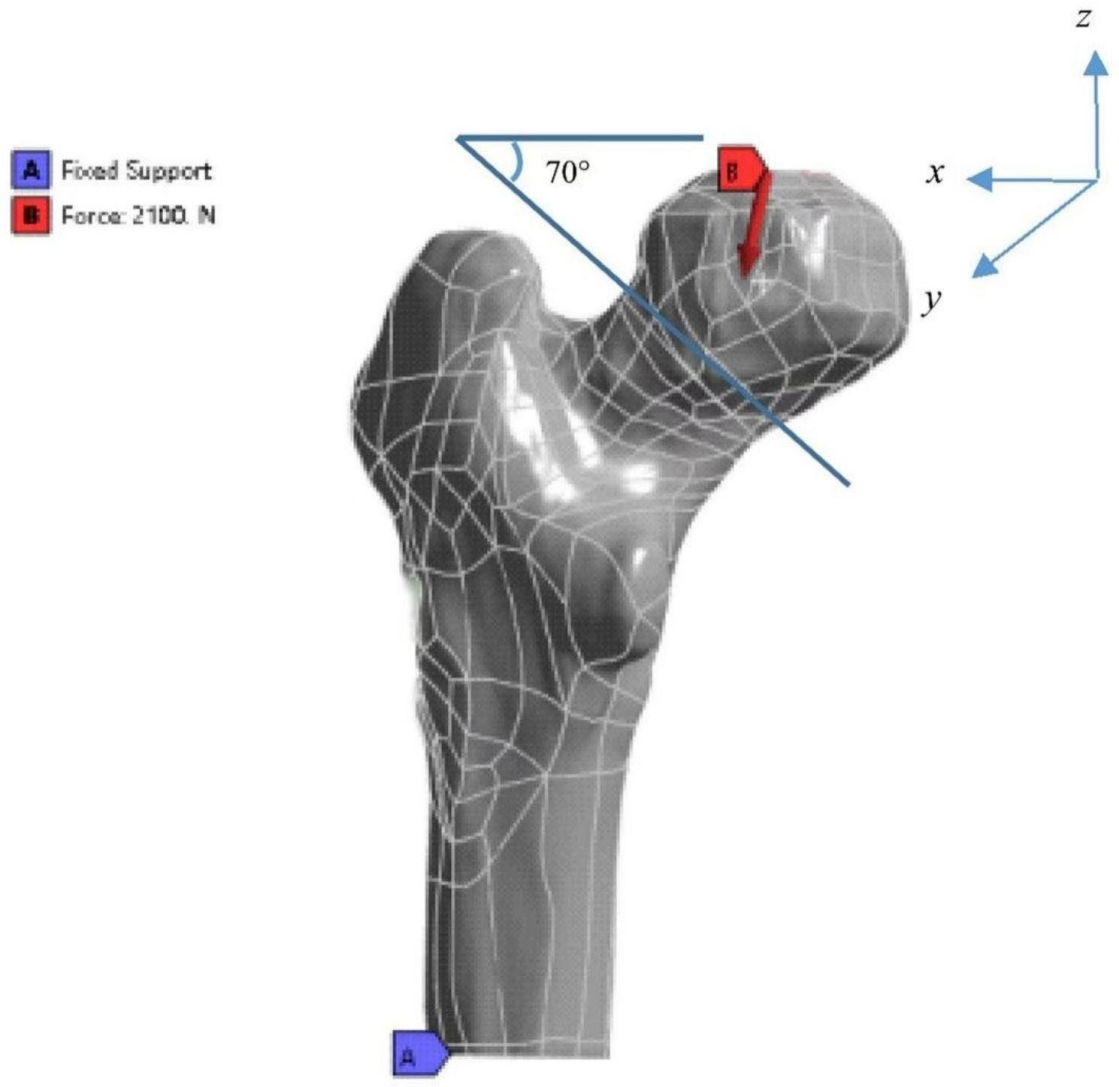
The model of Pauwels type III femoral neck fractures with the load vector of 2100 N

According to the characters of Pauwels type III femoral neck fractures, the included angle between the fracture line and the horizontal line is greater than 70 degrees; we created a plane through the center of the femoral neck that intersects the horizontal plane at an angle of 70 degrees to simulate Pauwels type III femoral neck fractures by using SolidWorks 2017 software (Dassault, France). (Fig. 3)

### Establishment of the model of internal fixation

**Fig. 4:**
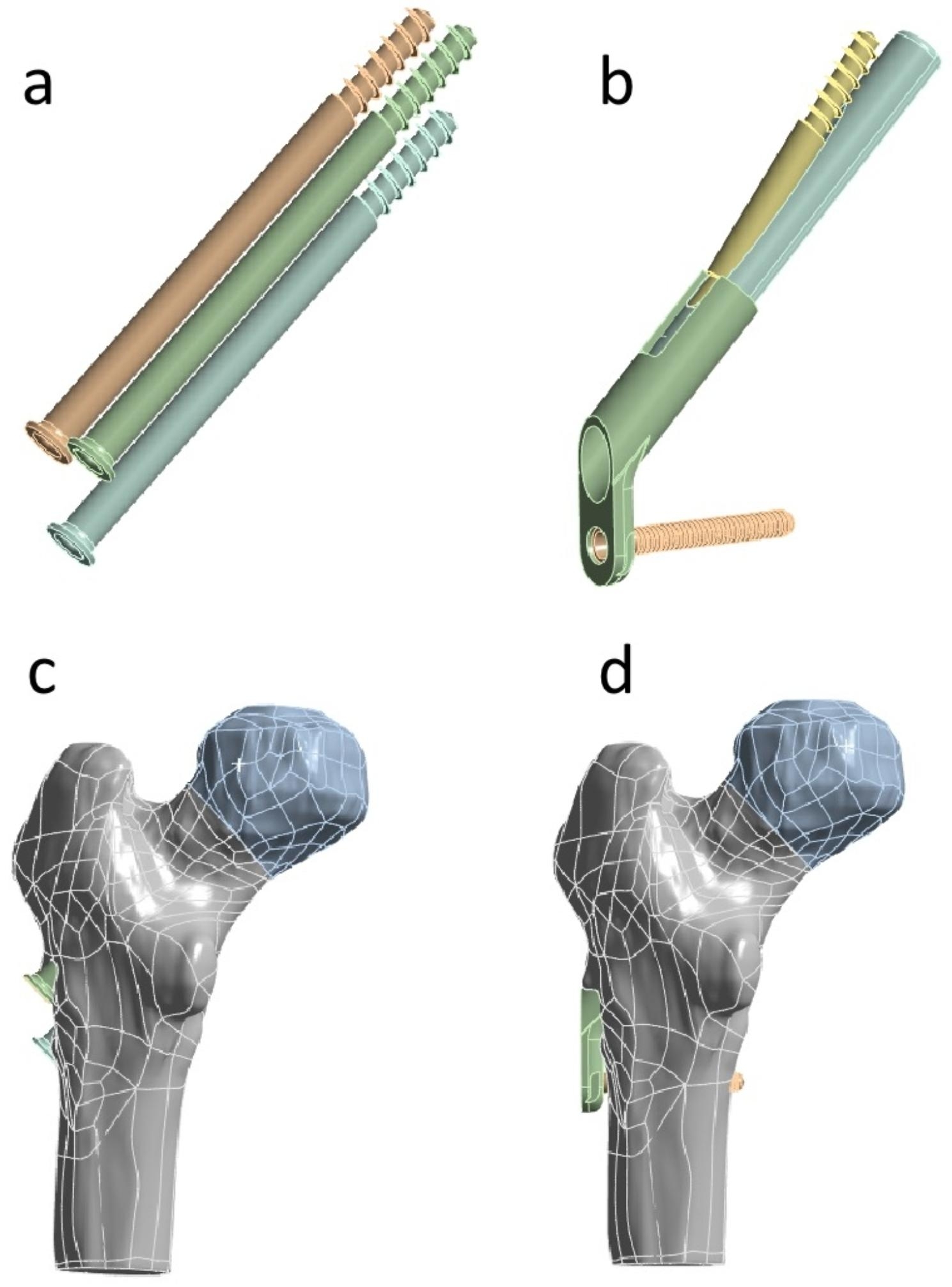
The model of internal fixation fixing Pauwels type III femoral neck fractures. (**a**) The model of three cannulated screws arranged in an inverted triangle. (**b**) The model of FNS. (**c**) The model of Pauwels type III femoral neck fractures fixated by three cannulated screws arranged in an inverted triangle. (**d**) The model of Pauwels type III femoral neck fractures fixated by FNS

SolidWorks 2017 software (Dassault, France) was used to establish the model of FNS and 3-CSs arranged in an inverted triangle. For the FNS model, the dynamic rod (sliding screw, 10 mm) was placed at an angle of 130 degrees to the locking plate with a 5.0-mm locking screw at the distal end, followed by the anti-rotational screw (6.4 mm) placed to the dynamic rod with an angle of 7.5 degrees. For the 3-CSs model, 3-CSs (7.3 mm) with partial thread were arranged in an inverted triangle parallel with each other, whose tips were located at 5 mm of the subchondral bone of the femoral head. Finally, Abaqus 2017 software (Simulia, France) was applied to mesh all models. (Fig. 4)

### Material parameters

All models applied in the study were considered continuous, isotropic, and uniform linear elastic materials, and the elastic modulus parameters of the femur and internal fixation devices are shown in Table 1.

**Table 1:** The elastic modulus parameters of the femur and internal fixation devices

Items	Femur		Titanium alloy
	Cortical bone	cancellous bone	
Elastic modulus (GPa)	16.8	0.84	105
Poisson’s ratio	0.3	0.2	0.35

### Contact settings, boundary conditions, and loading force settings

Referring to the previous studies [14, 15], there was a binding contact between the femur and internal fixation devices, and the connection between the fracture surface was set to friction with a friction coefficient of 0.3. Because the force loaded on the femur when standing on one leg was about three times the weight, the load vector of 2100 N was settled to the femoral neck, which inclined 12 degrees horizontally and 10 degrees backward based on the anteversion angle of the femur anatomy [16, 17].

### Evaluation criteria

In the finite element analysis, we analyze the data of deformation of the whole model, internal fixation, and fracture segments, the peak von Mises stress (VMS) of the whole model, internal fixation, distal femur, fracture segments, and contact at fracture segments including contact status and contact pressure in each group.

## Results

### Deformation

**Fig. 5:**
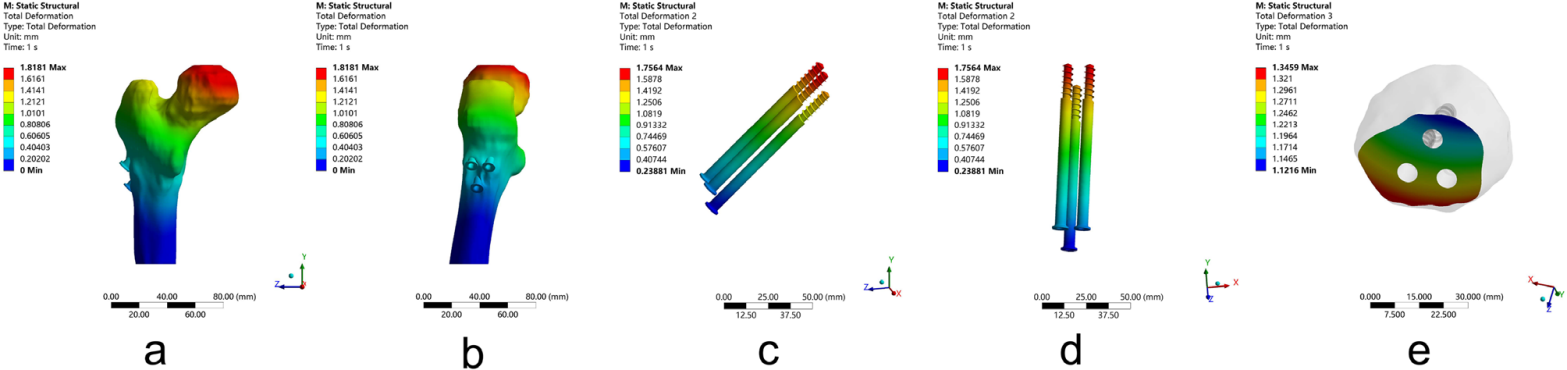
The deformation of femoral neck fractures fixated by three cannulated screws arranged in an inverted triangle. (**a**-**b**) The deformation of the whole model. (**c**-**d**) The deformation of the three cannulated screws. (**e**) The deformation of the fracture segment

**Fig. 6:**
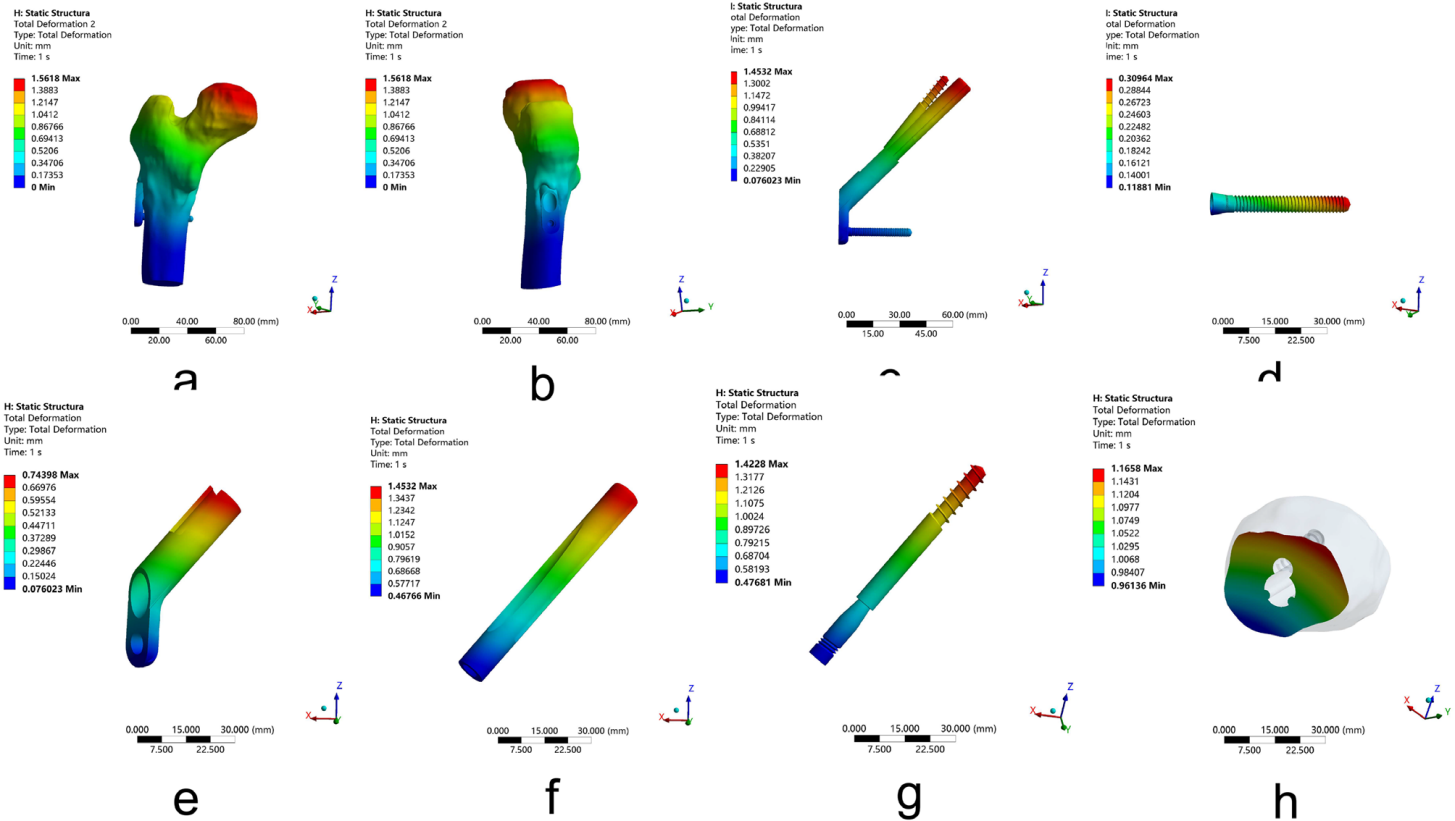
The deformation of femoral neck fractures fixated by FNS. (**a**-**b**) The deformation of the whole model. (**c**-**g**) The deformation of the FNS. (**h**) The deformation of fracture segments

For Pauwels type III femoral neck fractures, the total deformation of the whole model was located at the femoral head. After FNS fixation, the maximum deformation of the whole model was 1.56 mm, which is less than that of 3-CSs fixation, which was 1.82 mm. For FNS, the maximum deformation of internal fixation occurred at the top of the dynamic rod with 1.45 mm, and for 3-CSs arranged in an inverted triangle, the maximum deformation of internal fixation occurred at the upper two screws arranged parallel with 1.76 mm. After FNS fixation, the maximum deformation of the fracture segments is 1.17 mm, which is less than that of 3-CSs fixation, which is 1.35 mm. The results show that compared to 3-CSs, FNS can provide more stability to young Pauwels type III fractures with less deformation. (Figures 5 and 6; Table 2)

**Table 2:** The data of finite element analysis of FNS versus 3-CSs

		FNS	3-CSs
Deformation (mm)	whole model	1.56	1.82
	internal fixation	1.45	1.76
	fracture segments	1.17	1.35
Peak VMS (MPa)	whole model	108.63	86.03
	distal femur	54.77	66.16
	internal fixation	108.63	86.03
	fracture segments	40.74	48.99
Contact at fracture segments	contact status	Sticking on the middle and lower 1/3 segment of femoral neck	Sticking on the middle and lower 1/3 segment of femoral neck
	contact pressure (MPa)	8.95	11.68

### VMS

**Fig. 7:**
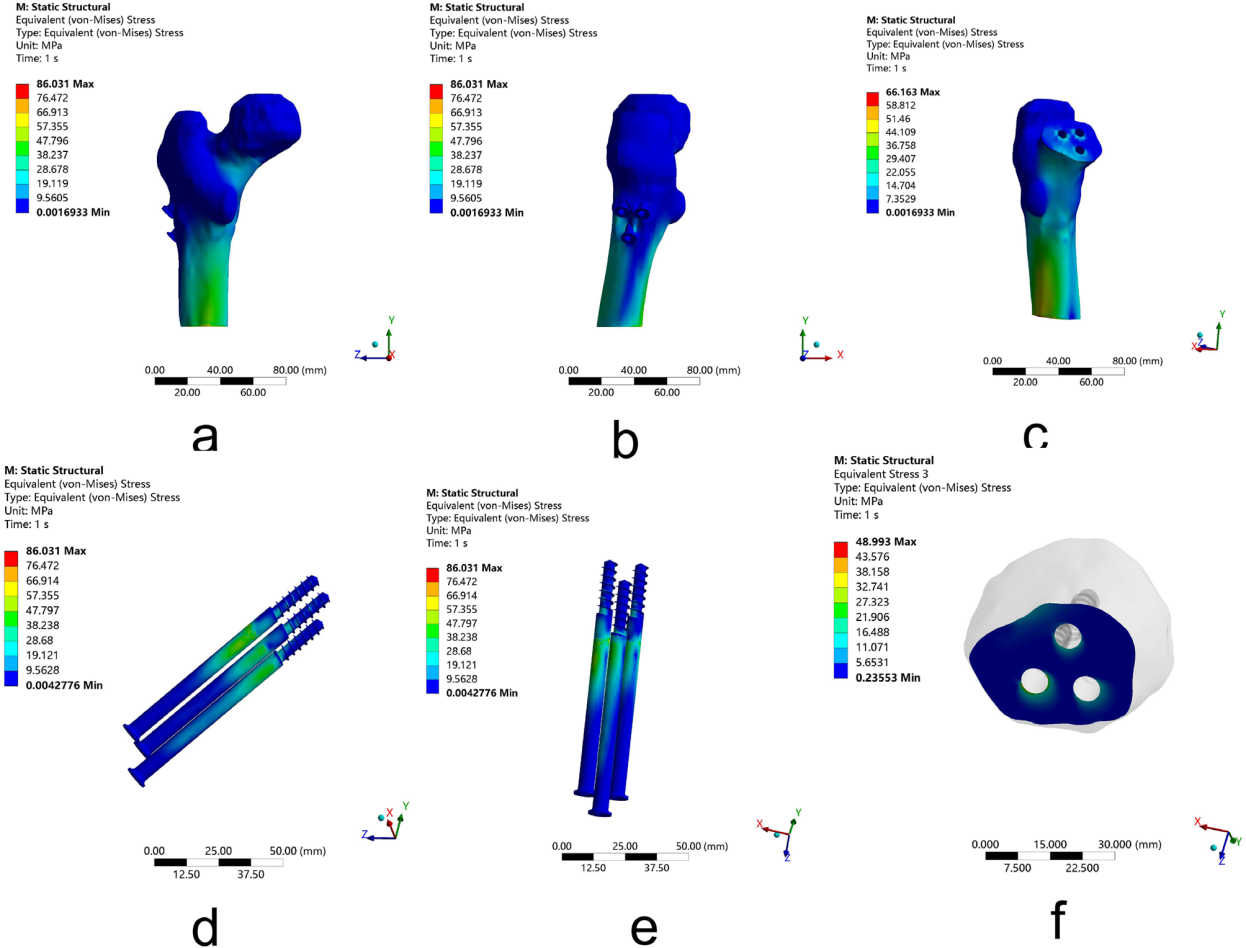
The VMS of femoral neck fractures fixated by three cannulated screws arranged in an inverted triangle. (**a**-**b**) The peak VMS of the whole model. (**c**) The peak VMS of the distal femur. (**d**-**e**) The peak VMS of three cannulated screws arranged in an inverted triangle. (**f**) The peak VMS of the fracture segment

**Fig. 8:**
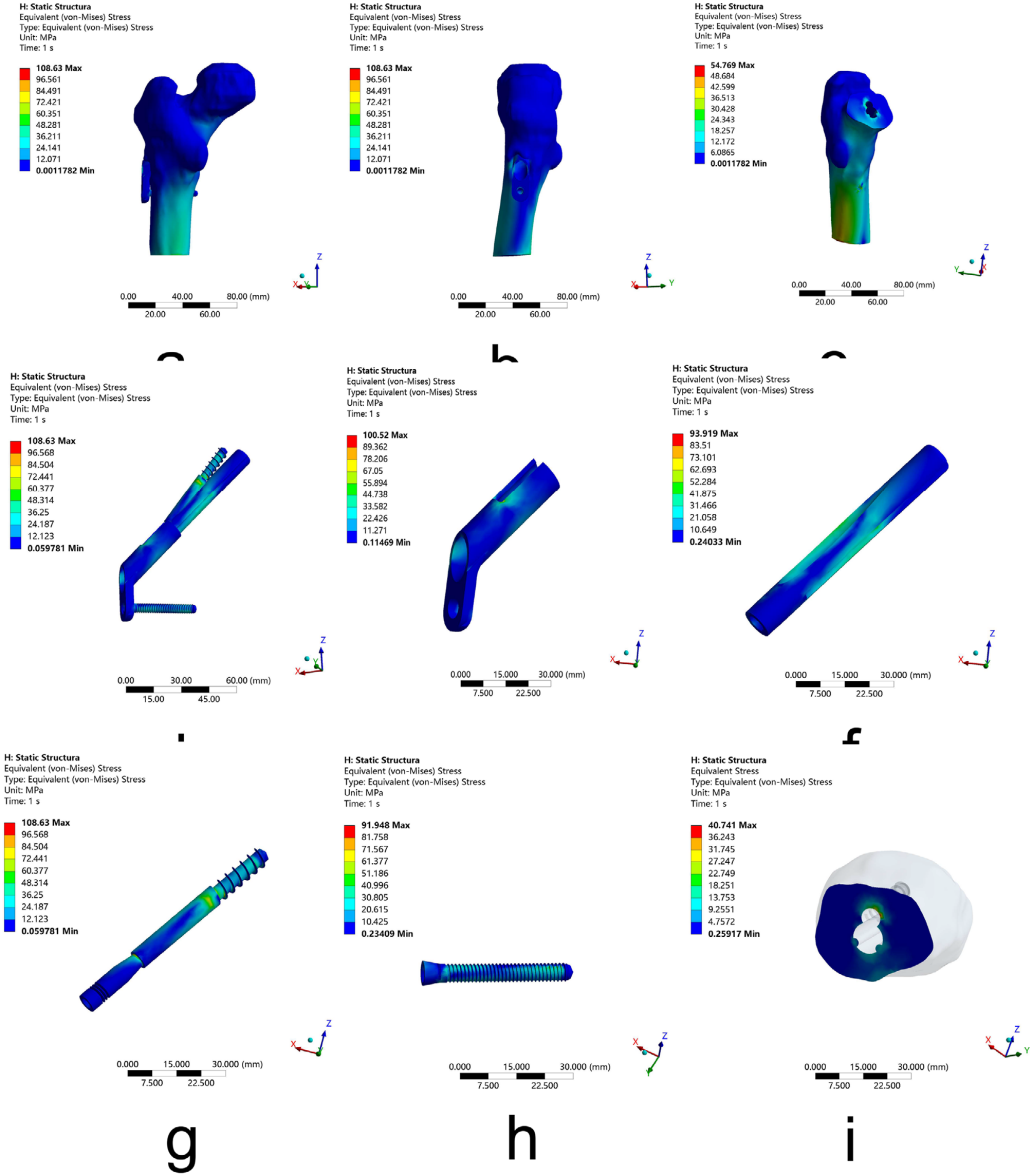
The VMS of femoral neck fractures fixated by FNS. (**a**-**b**) The peak VMS of the whole model. (**c**) The peak VMS of the distal femur. (**d**-**i**) The peak VMS of FNS. (**j**) The peak VMS of the fracture segment

Under the young Pauwels type III femoral neck fractures condition, the peak VMS of the whole model appeared to concentrate at the inside of the model with 108.63 MPa after FNS fixation and 86.03 MPa after 3-CSs fixation. The VMS of the femur was analyzed, and the peak VMS of the distal femur and fracture segments were 54.77 MPa and 40.74 MPa separately after FNS fixation and 66.16 MPa and 48.99 MPa separately after 3-CSs fixation. While the peak VMS of internal fixation components is 108.63 MPa for FNS, which is concentrated at the root of the thread of the anti-rotation screw, and 86.03 MPa for 3-CSs, which is concentrated at the upper front screw. The results indicate that for young Pauwels type III femoral neck fractures, although there is higher stress on the whole model and internal fixation components after FNS fixation, FNS can provide lower stress on the distal femur and fracture segments compared to 3-CSs fixation. (Figures 7 and 8; Table 2)

### Contact status and contact pressure

**Fig. 9:**
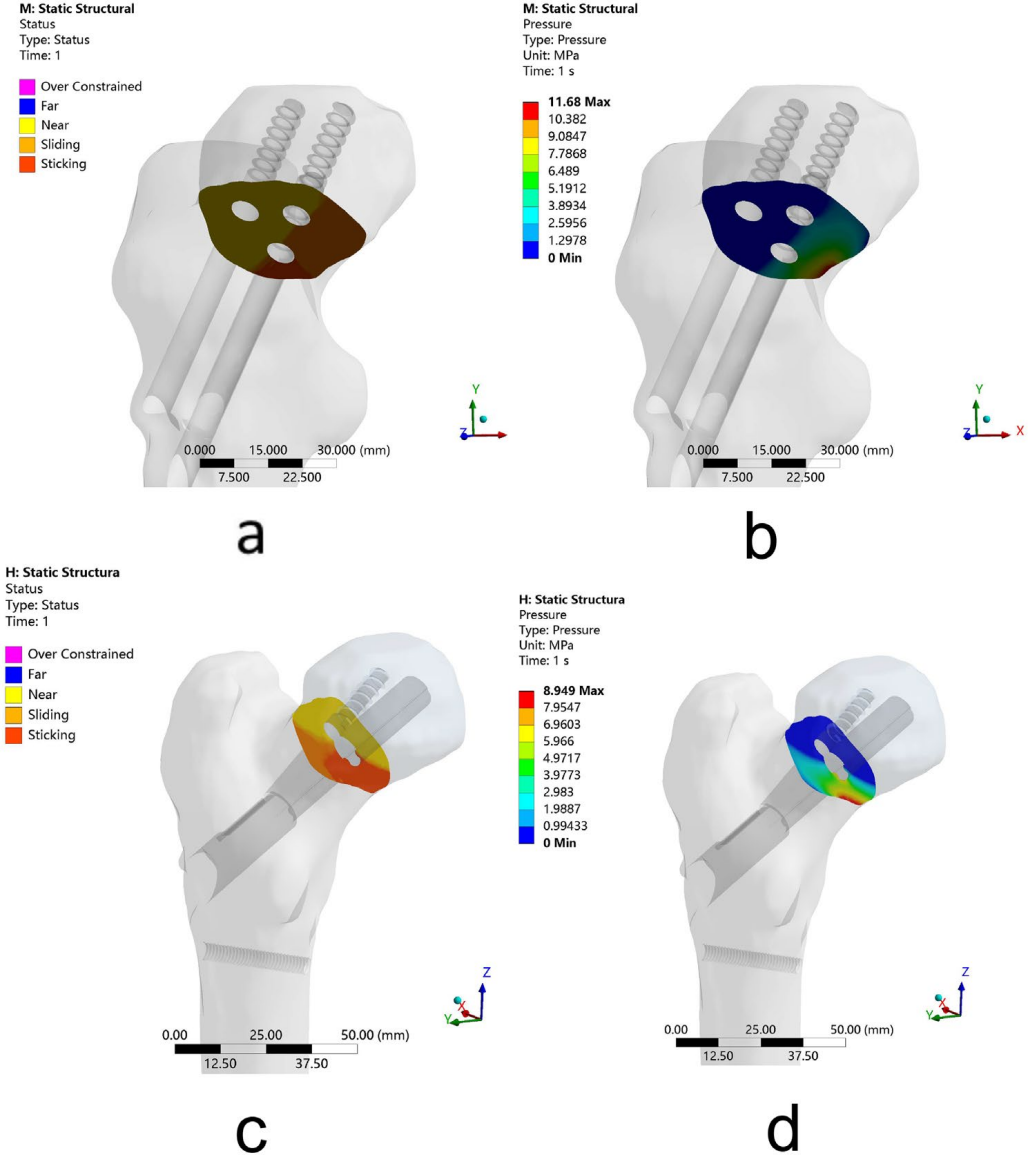
The contact at fracture segments after internal fixation. (**a**) The contact status of fractures after three cannulated screws arranged in an inverted triangle fixation. (**b**) The contact pressure of fractures after three cannulated screws arranged in an inverted triangle fixation. (**c**) The contact status of fractures after FNS fixation. (**d**) The contact pressure of fractures after FNS fixation

After FNS fixation, the contact status of the fracture fragments is sticking at the middle and lower 1/3 segment of the femoral neck with the 8.95 MPa maximum contact pressure. While after 3-CSs fixation, the contact status of the fracture fragments is also sticking at the middle and lower 1/3 segment of the femoral neck, but the maximum contact pressure is 11.68 MPa. The results show that, with the same contact status at young Pauwels type III femoral neck fractures, FNS can produce less contact pressure than 3-CSs between fracture segments (Fig. 9; Table 2).

## Discussion

It is crucial to reduce fractures anatomically and maintain fracture stability for young patients with Pauwels III femoral neck fractures so that internal fixation devices with mechanical stability are necessary for satisfactory clinical effects of femoral neck fractures [1]. Because of the high shear force and shear stress, it is difficult to maintain the stability of Pauwels III femoral neck fractures by internal fixation devices, and it is still a medical conundrum which internal fixation devices are optimal for young femoral neck fractures [18, 19]. 3-CSs have been widely used for fixing femoral neck fractures in clinics. Following the theory of “sliding compression”, 3-CSs arranged in an inverted triangle can not only provide mechanical support but also form a sliding track. Under the contraction of the hip muscles, the fracture blocks can slide along the axis of the femoral neck, forming pressure at the broken end of the fracture to promote fracture healing [20]. Although 3-CSs can provide mechanical stability to femoral neck fractures, studies [21–23] showed that this internal fixation had a high risk of bone nonunion (incidence 7.4 − 19%), femoral head necrosis (incidence 11.5 − 14.3%) and internal fixation failure (incidence 9.7 − 13.1%). Besides, some studies [13, 24, 25] found a high risk of femoral neck shorting and coxa vara for patients with femoral neck fractures treated by 3-CSs arranged in an inverted triangle. So, it is not enough to resist the shear force of femoral neck fracture for 3-CSs arranged in an inverted triangle without a clear biomechanical mechanism, and there are many reasons for it. On the one hand, the sliding compression force perpendicular to the fracture line is only a partial force of the hip joint force, and the other partial force increases the shear stress on the fracture and internal fixation devices, causing fracture displacement and fixation failure [15]. On the other hand, the present study has shown that there are worse deformations of the whole model, internal fixation, and fracture segments after 3-CSs arranged in an inverted triangle fixation meaning that 3-CSs cannot provide enough mechanical stability for young Pauwels type III femoral neck fractures which is similar to the previous study.

As a modified implant of the dynamic hip screw (DHS), FNS consists of three parts (dynamic rod, anti-rotation screw, and locking plate) with smaller volume, simpler operation, and less trauma to provide angle stability and can reduce the destruction of the blood supply to the femoral head [11, 13]. Every part of FNS plays an important role in treating femoral neck fractures. The dynamic rod is a cylindrical design that provides angular stability, and the anti-rotation screw provides rotary stability, which can slide together with a dynamic rod up to 20 mm to compress fractures. With minimal implant trace design, the plate can provide angular stability to fractures. Nevertheless, as a newly developed implant, the time of FNS applied in clinics is too short to evaluate its effects on femoral neck fractures, and there are fewer bio-mechanical studies to show the mechanical properties of FNS and its mechanical mechanism on fixing young Pauwels III femoral neck fractures is still unknown.

To further realize the biomechanical effects and mechanism of FNS for young patients with Pauwels III femoral neck fractures, we performed the finite element analysis. Results found that for young patients with Pauwels III femoral neck fractures, compared to 3-CSs arranged in an inverted triangle, there are fewer deformations of the whole model, internal fixation, and fracture segments of FNS fixation, meaning that FNS can provide better mechanical stability for young patients with Pauwels III femoral neck fractures and the results are similar to the previous study [26]. The reason may be related to the effect of each part of the FNS and the 7.5-degree angle between the anti-rotation screw and dynamic rod, and bio-mechanical studies [27, 28] carried out on animal carcasses also indicated that the mechanical properties of FNS, including mechanical stability and rotary stability, were better than that of hansson pins, DHS, and cannulated screws. Besides, we analyzed the contact at fracture segments of FNS and 3-CSs arranged in an inverted triangle separately, finding that compared to 3-CSs, FNS can provide sticking contact to fracture segments with less contact pressure on the middle and lower 1/3 segment of the femoral neck which may be benefited from sliding compression of the dynamic rod. Because the femoral neck shorting and coxa vara caused by bone resorption is related to the contact pressure and contact status, better contact status and less contact pressure may be the reasons that the incidence of femoral neck shorting and coxa vara of FNS for femoral neck fractures is less than that of 3-CSs. Although some clinical studies [12, 13] reported that the incidence of internal fixation failure of FNS is less than 3-CSs, the present study showed that for young Pauwels type III femoral neck fractures, the stress of whole model and internal fixation of FNS was higher than 3-CSs and the stress of FNS device was mainly concentrated on the root of the thread on anti-rotation screw and the junction of the plate and locking screw meaning that FNS can be broken easily on these positions which cannot explain the conclusion of previous clinical studies [12, 13]. However, the peak VMS of the distal femur and fracture segments after FNS fixation was lower than 3-CSs, indicating that the stress of FNS on the femur, especially on the fracture segments, is low, which is helpful to bone healing and related to the high incidence of bone healing of FNS.

Besides, the latest study [29] has reported that there is a risk of peri-implant subtrochanteric femur fractures in patients with incomplete non-displaced fractures who received FNS fixation. Our study found that the peak VMS of the locking plate and locking screw in FNS around the lesser trochanter is about 80 MPa which is enough to cause a stress fracture. Hence, the design of locking screws in the plate not only increases construct stiffness but also causes stress concentration which may lead to peri-implant subtrochanteric femur fractures. In our opinion, the locking plate of FNS should be extended with a distal hole placed on the distal to the lesser trochanter, which may be helpful in decreasing the risk of peri-implant subtrochanteric femur fractures.

Compared to previous studies [30] about DHS or DHS combined with additional lag screws on treating femoral neck fracture, the advantages of FNS on fixing femoral neck fracture include integrated design providing construct stiffness to avoid internal fixation failure and small volume, which is helpful to achieve mini-invasive implantation and decrease irritation to soft tissue and skin.

Compared to the proximal femoral nail (PFN), another commonly used implant for femoral neck fracture, both FNS and 3-CSs have biomedical advantages. Compared to a previous study [31], the deformation or displacement of fracture segments after FNS fixation and 3-CSs are both shorter than that of PFN fixation. Besides, no matter where the PFN is placed, the peak VMS of PFN is significantly stronger than that of FNS and 3-CSs. Therefore, both FNS 3-CSs can provide more stability and less stress to femoral neck fracture than PFN, which has superior construct stiffness.

However, there are some limitations of the present study. Firstly, we only comparatively analyze the bio-mechanical effects and mechanism of FNS and 3-CSs arranged in an inverted triangle on young patients with Pauwels type III femoral neck fractures without old patients and Pauwels type I and II femoral neck fractures. Secondly, we only compared the biomechanical effects of FNS and 3-CSs arranged in an inverted triangle in the fixation of young patients with Pauwels type III femoral neck fractures, not DHS and other internal fixations. Lastly, the results of the finite element analysis are inevitably different from the actual results.

In a word, compared to 3-CSs arranged in an inverted triangle, FNS can provide better mechanical effects such as more mechanical stability, lower stress on fracture segments, and sticking contact status with less contact pressure for young patients with Pauwels type III femoral neck fractures which may be the mechanical mechanism of clinical effects of FNS on femoral neck fracture. Although there is high stress on FNS, it is still an effective and safe internal fixation for young patients with Pauwels type III femoral neck fractures.

## Data Availability

Data will be available from the corresponding author on rationale request.
